# Supplementary figures and images for: Single-step generation of homozygous knockout/knock-in individuals in an extremotolerant parthenogenetic tardigrade using DIPA-CRISPR
Source: PLoS Genet. 2024 Jun 13;20(6):e1011298. doi: 10.1371/journal.pgen.1011298 (PMC11175437; doi:10.1371/journal.pgen.1011298)

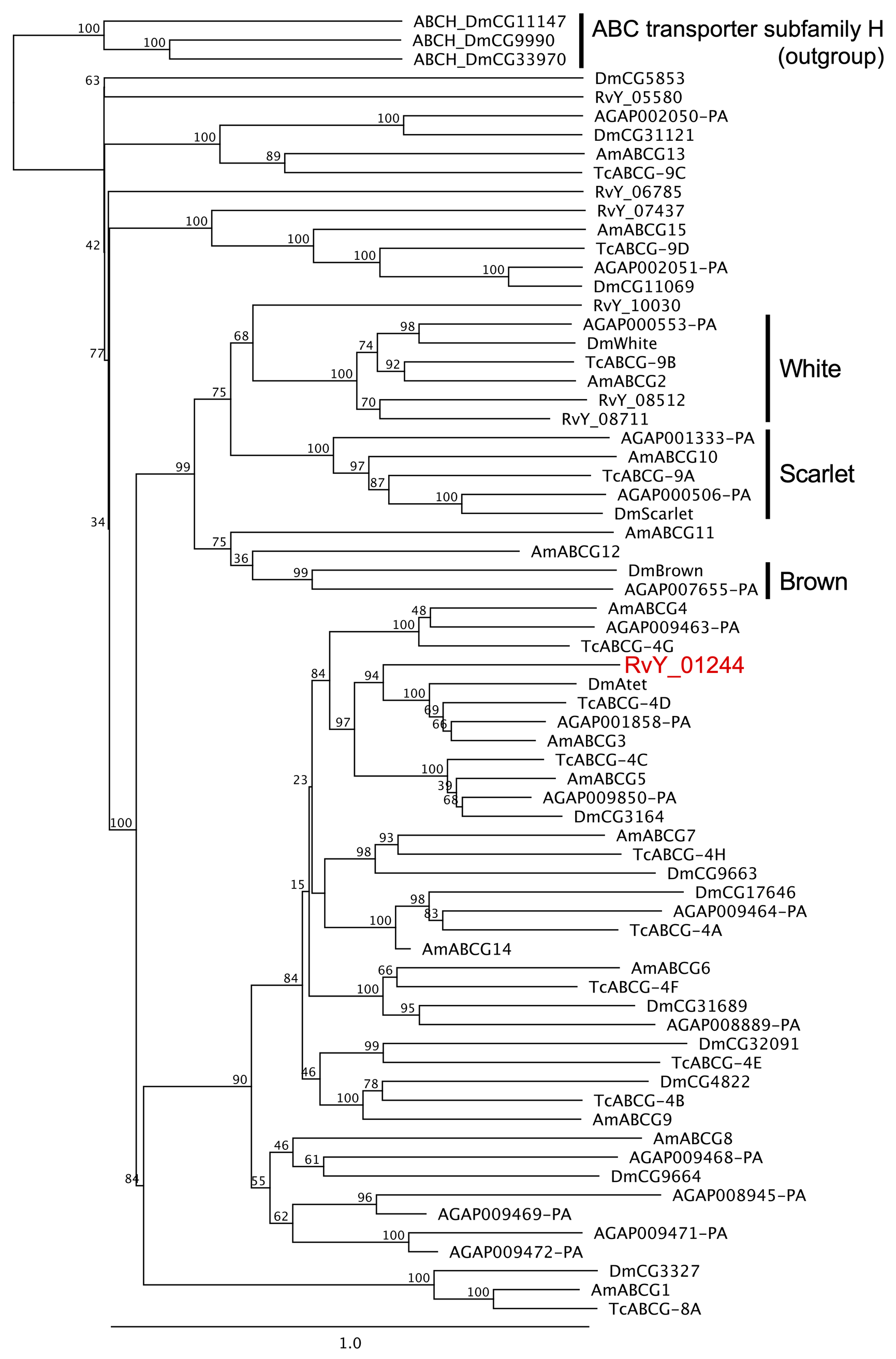

Supplement: S1 Fig — Phylogenetic analysis using 66 ABCG proteins from a tardigrade (Rv, Ramazzottius varieornatus) and four insects (AG, Anopheles gambiae; Am, Apis mellifera; Dm, Drosophila melanogaster; Tc, Tribolium castaneum). Maximum likelihood analysis was performed with the substitution model LG. Red indicates the gene targeted by CRISPR-Cas9 in this study. Three proteins of ABC transporter subfamily H were used as an outgroup. (TIF) [file pgen.1011298.s001.tif]

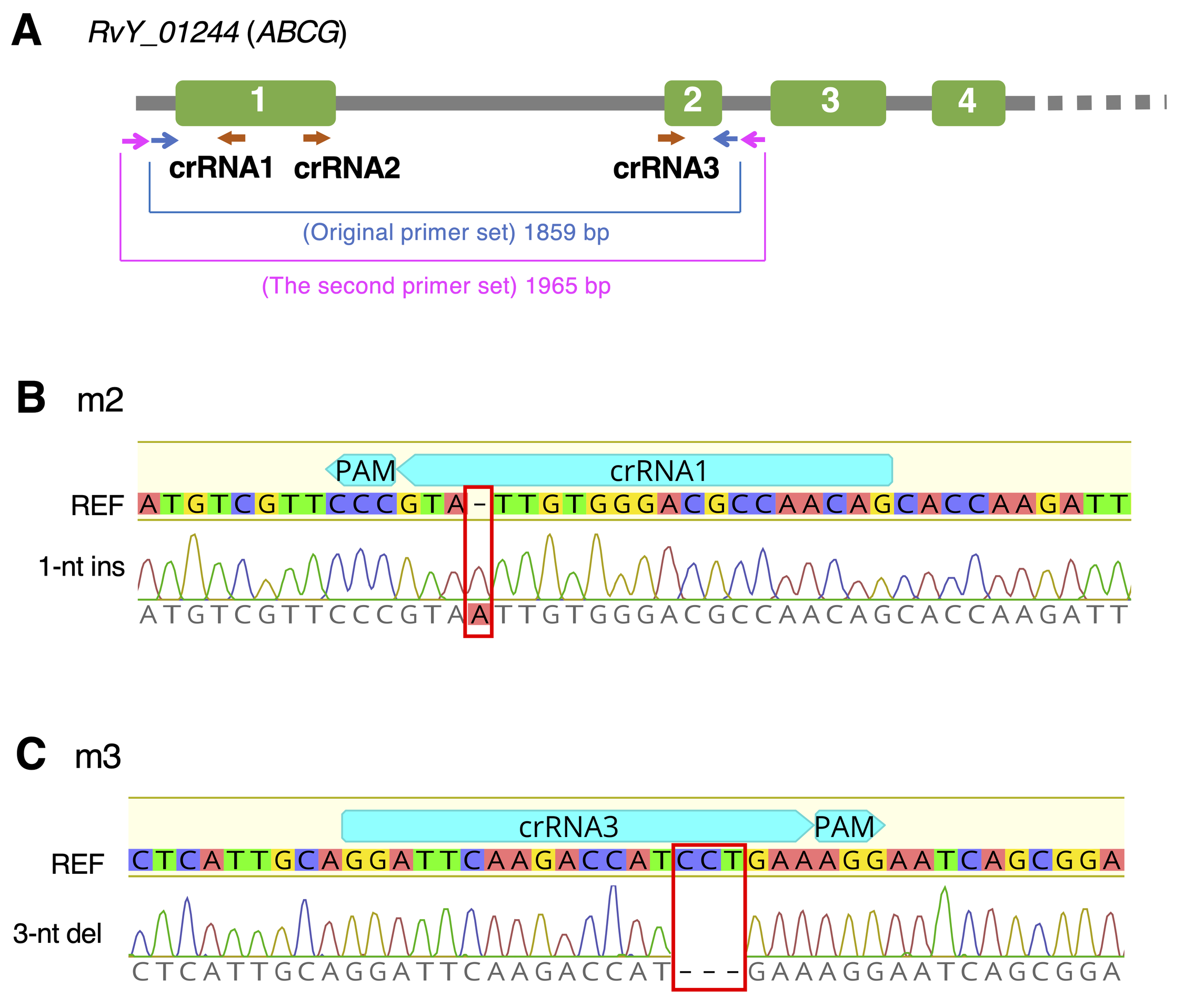

Supplement: S2 Fig — (A) Schematic representation of the second primer set (magenta arrows) with the structure of the RvY_01244 (ABCG) gene, three crRNAs (brown arrows), and the original set of PCR primers (blue arrows). Green boxes represent exons and gray lines represent introns or intergenic regions. (B, C) Electropherograms in direct Sanger sequencing of genomic PCR amplicons amplified with the second primer set. (B) m2 carrying a 1-nt insertion. (C) m3 carrying a 3-nt deletion. No mixed peaks were detected, suggesting that these edits were present homozygously. (TIF) [file pgen.1011298.s002.tif]

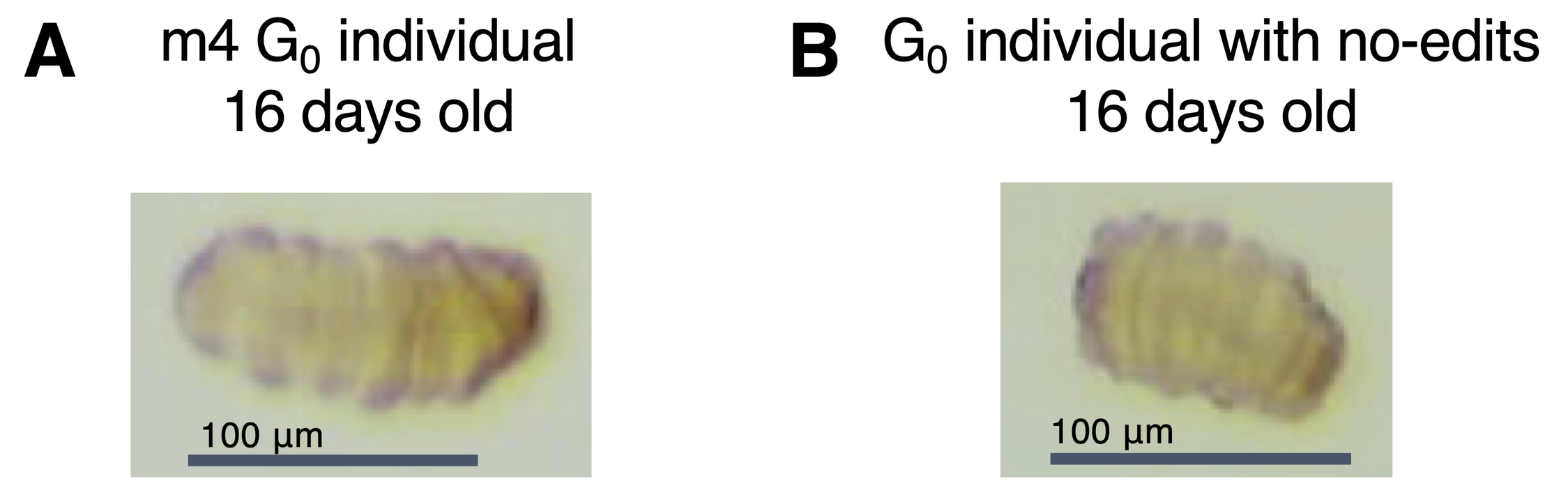

Supplement: S3 Fig — Photographs of G0 individuals carrying the m4 mutation (A) and no edits (B) at 16 days old. The m4 mutant exhibited a brown color and was apparently indistinguishable from the G0 individual carrying no edits. Compared with wild-type individuals (Fig 1B and 1C), both of them were much smaller and had a relatively faint body color. (TIF) [file pgen.1011298.s003.tif]

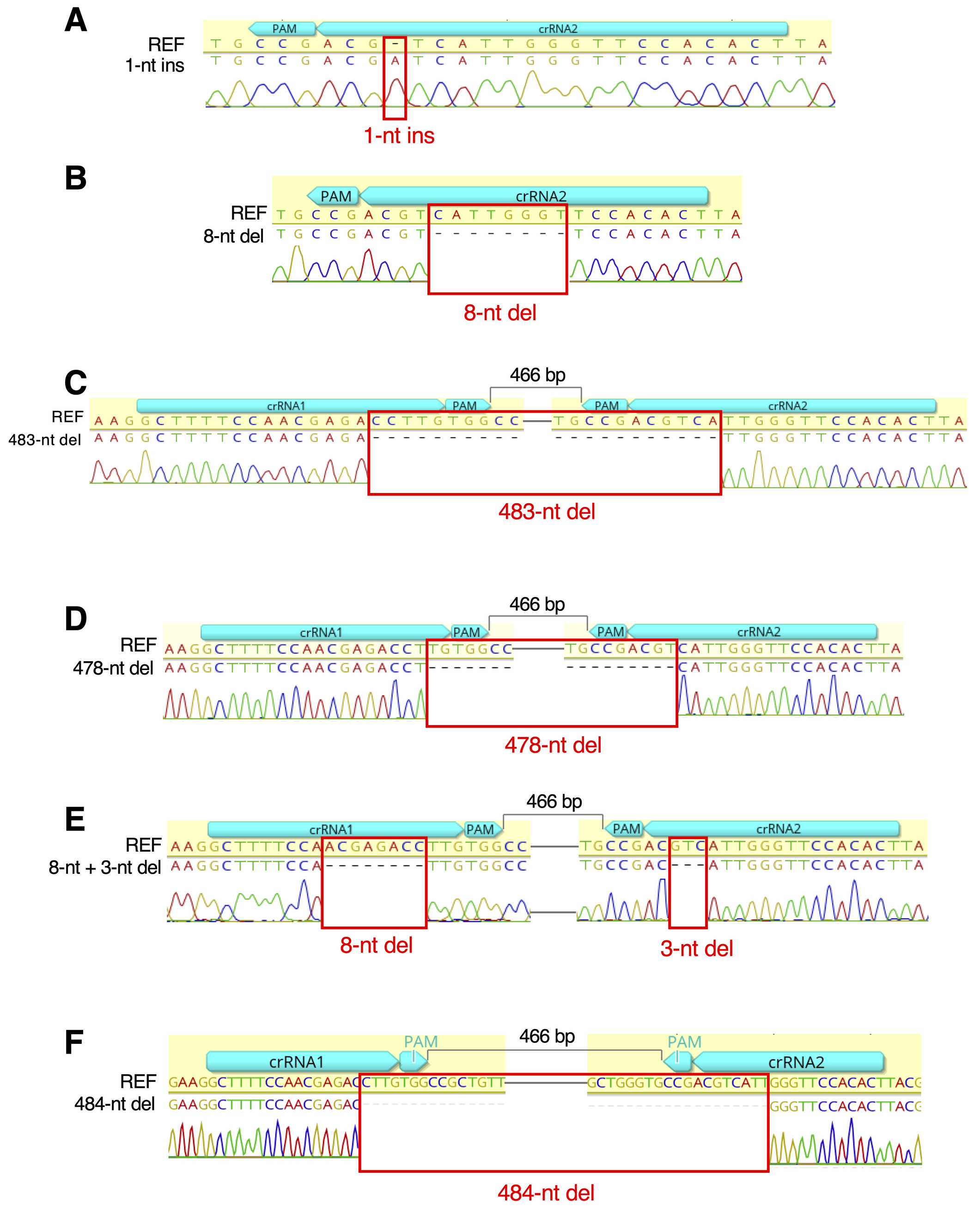

Supplement: S4 Fig — Electropherogram data corresponding to Fig 3C (A, 1-nt ins; B, 8-nt del; C, 483-nt del; D, 478-nt del) and Fig 3E (E, 8-nt + 3-nt del; F, 484-nt del). No mixed peaks were detected, suggesting that all of these G0 individuals were homozygous mutants. (TIF) [file pgen.1011298.s004.tif]

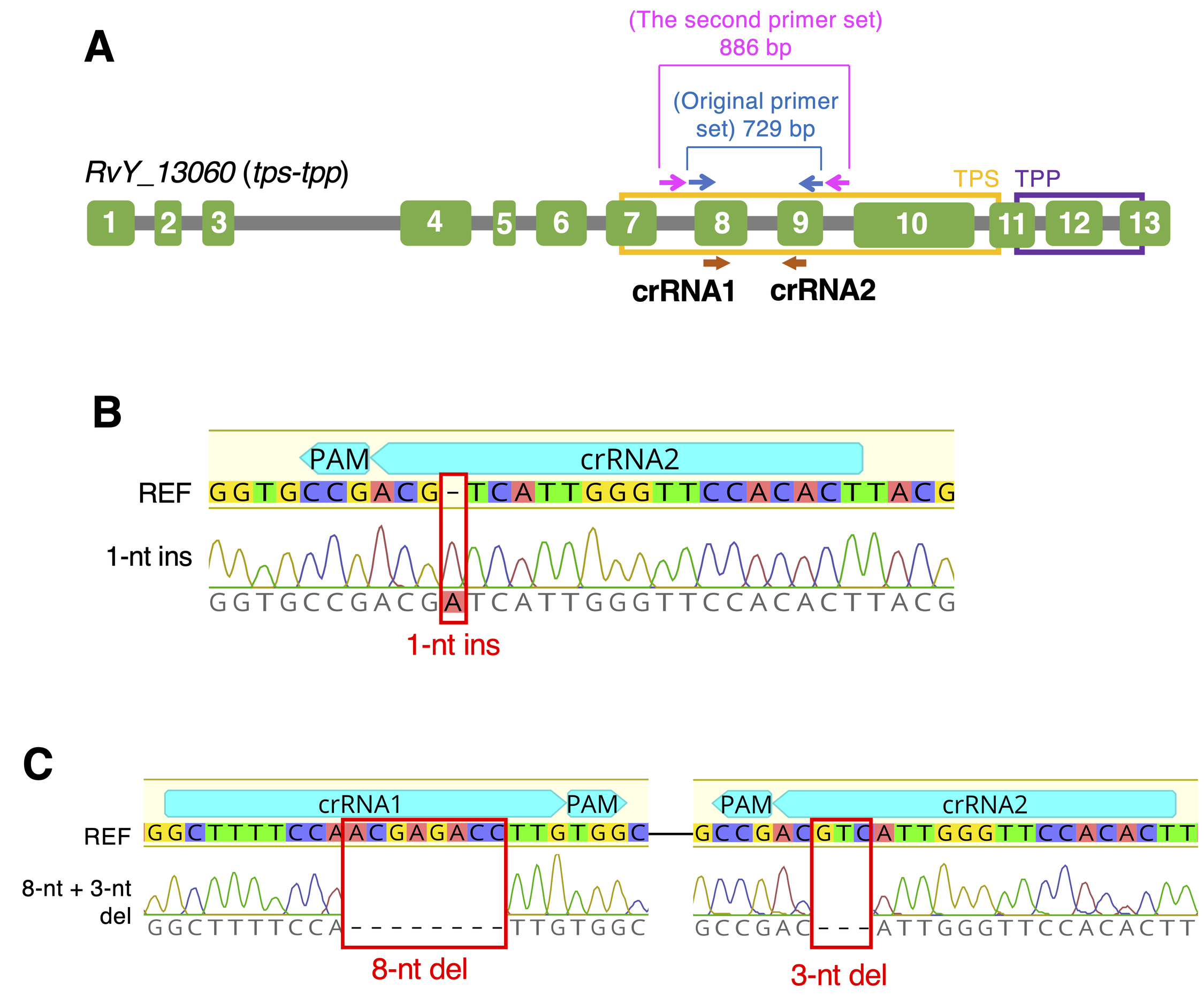

Supplement: S5 Fig — (A) Schematic representation of the second primer set (magenta arrows) with the structure of the RvY_13060 (tps-tpp) gene, two crRNAs (brown arrows), and the original set of PCR primers (blue arrows). Green boxes represent exons and gray lines represent introns. (B, C) Electropherograms in direct Sanger sequencing of the genomic PCR amplicons amplified with the second primer set. Data correspond to Fig 3E (B, 1-nt ins; C, 8-nt + 3-nt del). No mixed peaks were detected, suggesting that these edits were present homozygously. (TIF) [file pgen.1011298.s005.tif]

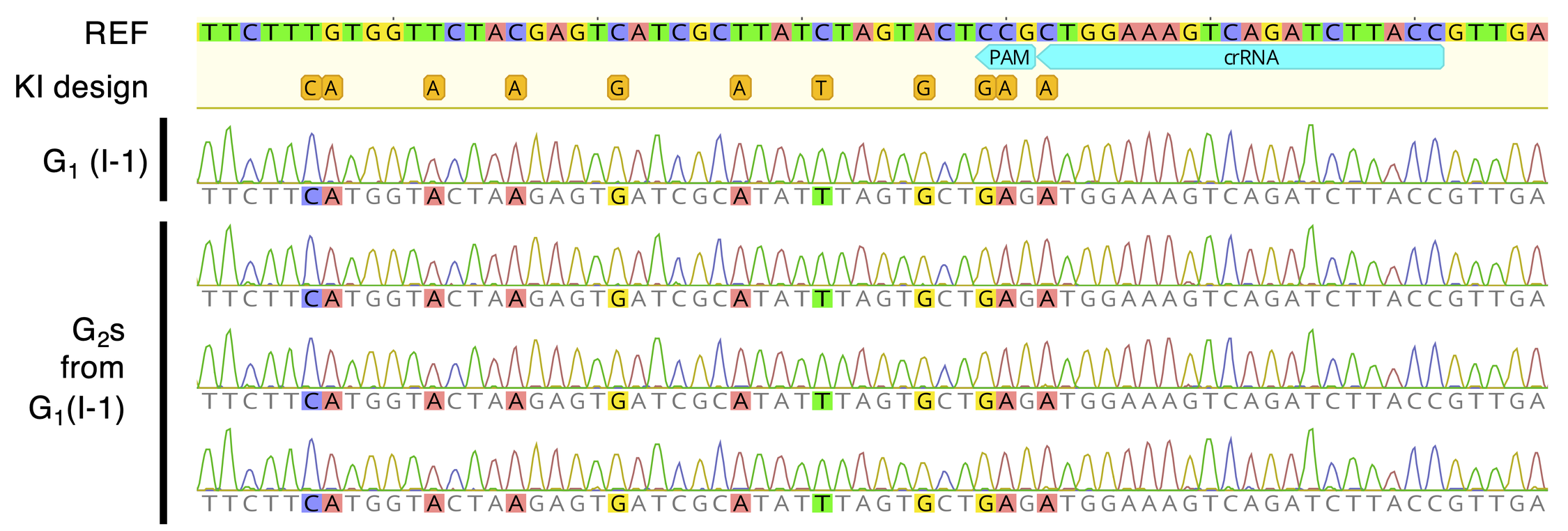

Supplement: S6 Fig — Representative electropherograms of a G1 individual (I-1) and three G2 individuals derived from a perfectly knocked-in G0 individual (I; S5 Table). REF represents the sequence of the unmodified genome, and the designed substitutions in ssODNs are shown as a KI design. The substituted bases are shown highlighted. All progeny exhibited clear single sequences without mixed peaks, indicating the heritability of the knock-in sequence as a homozygous mutation. (TIF) [file pgen.1011298.s006.tif]

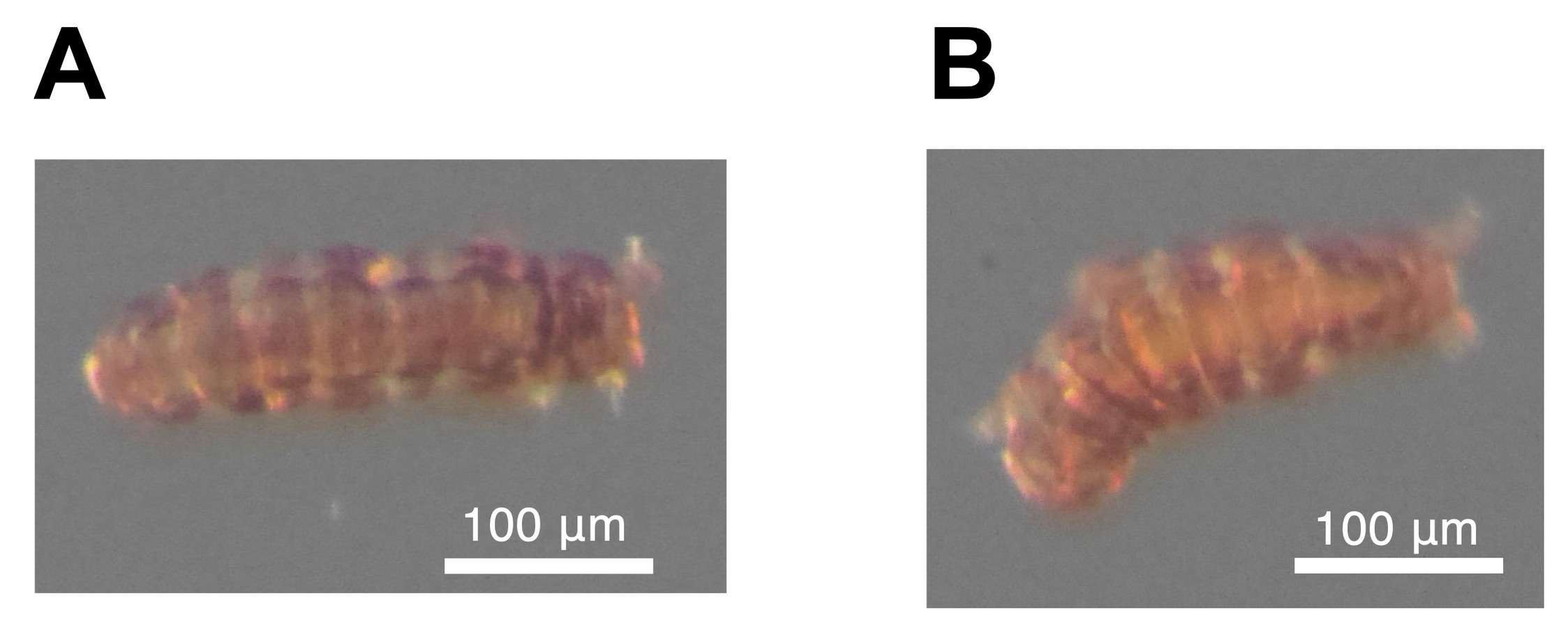

Supplement: S7 Fig — Representative photographs of a knock-in individual carrying all of the 11 substitutions in the RvY_01244 (ABCG) gene (perfect substitutions; A) and an individual carrying no edits (B). The body color of knock-in individuals appeared comparable to that of those with no edits. (TIF) [file pgen.1011298.s007.tif]

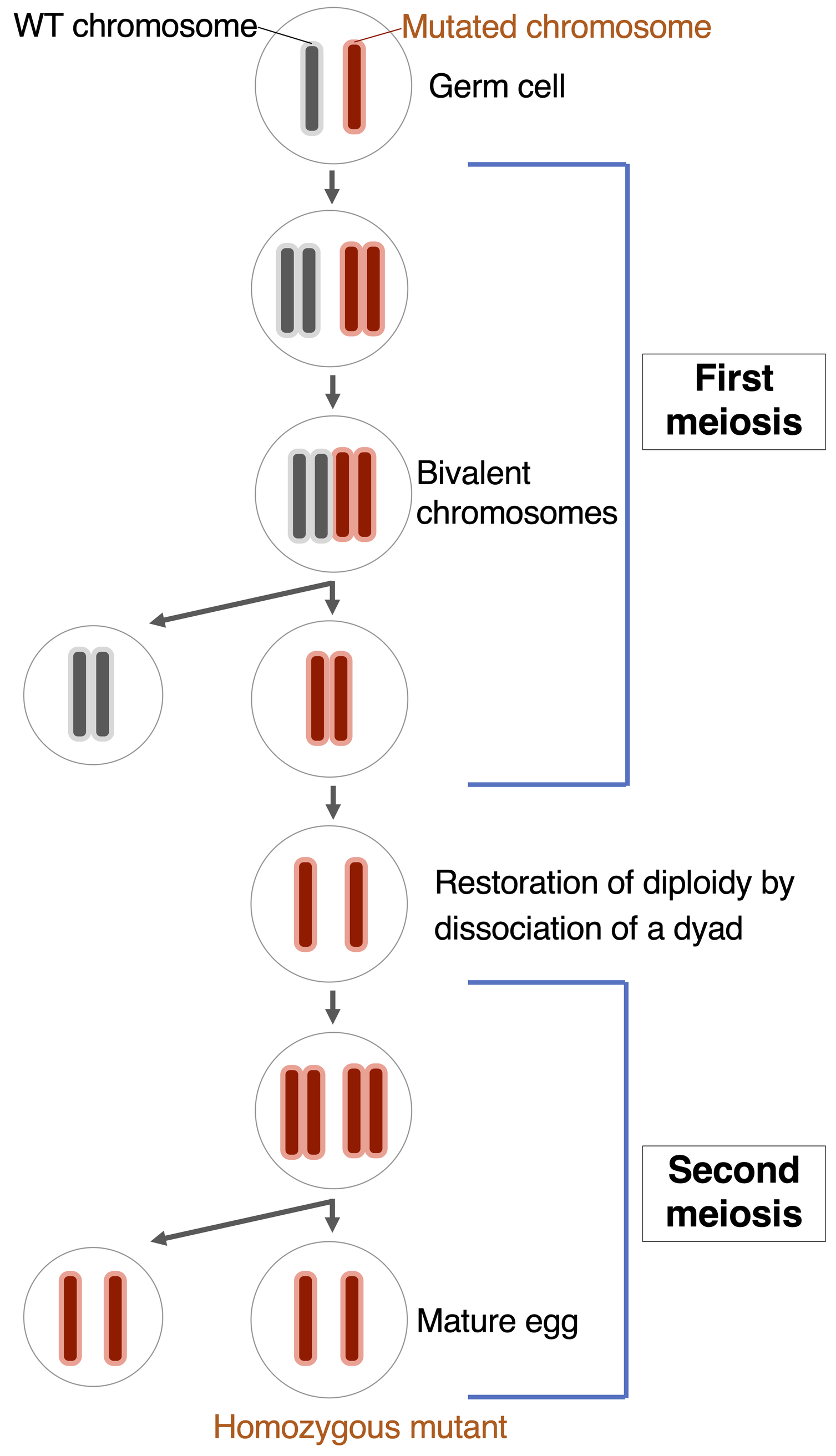

Supplement: S8 Fig — (TIF) [file pgen.1011298.s008.tif]

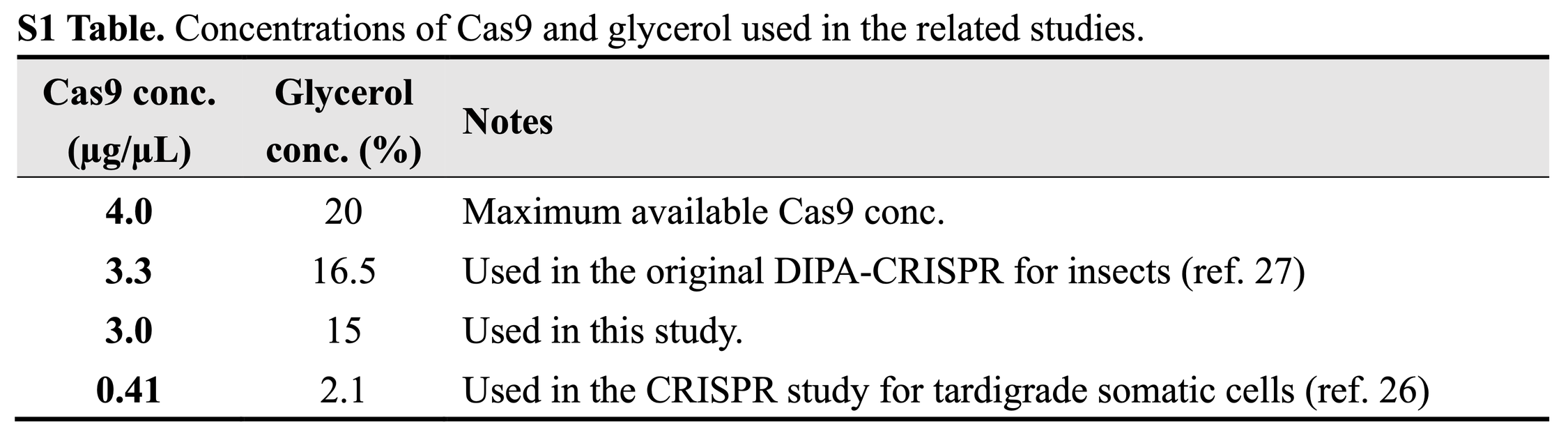

Supplement: S1 Table — (TIF) [file pgen.1011298.s009.tif]

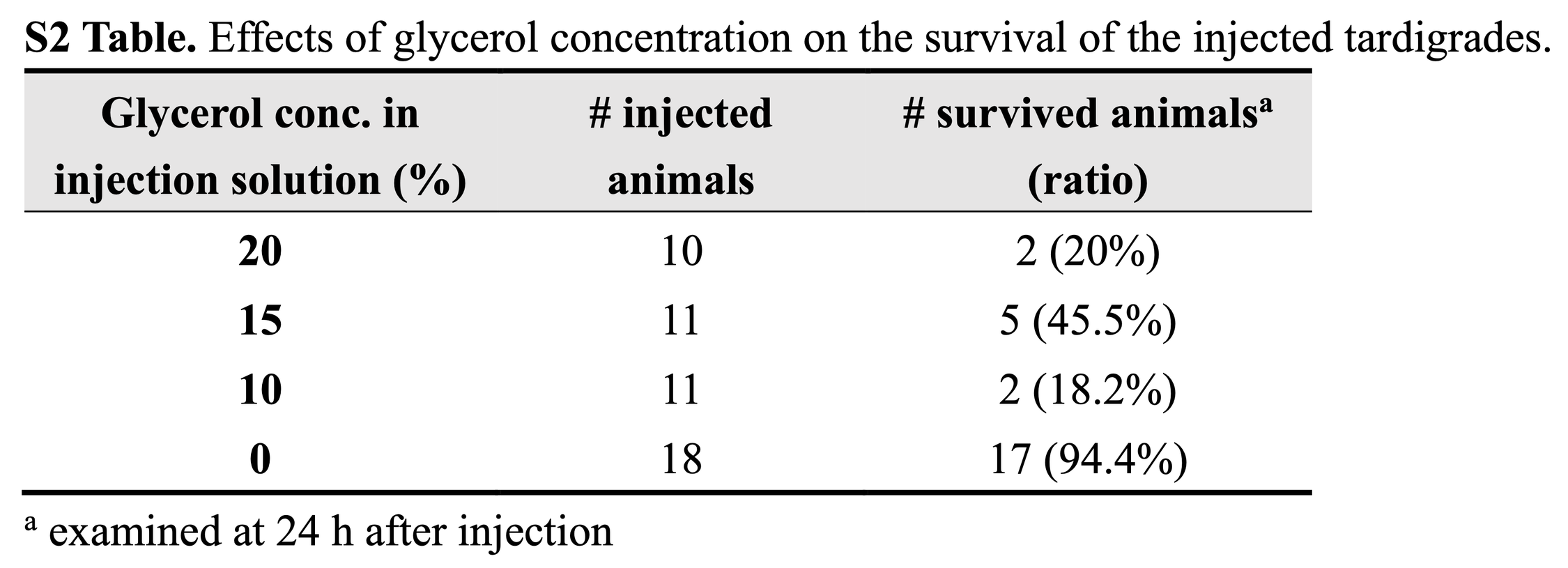

Supplement: S2 Table — (TIF) [file pgen.1011298.s010.tif]

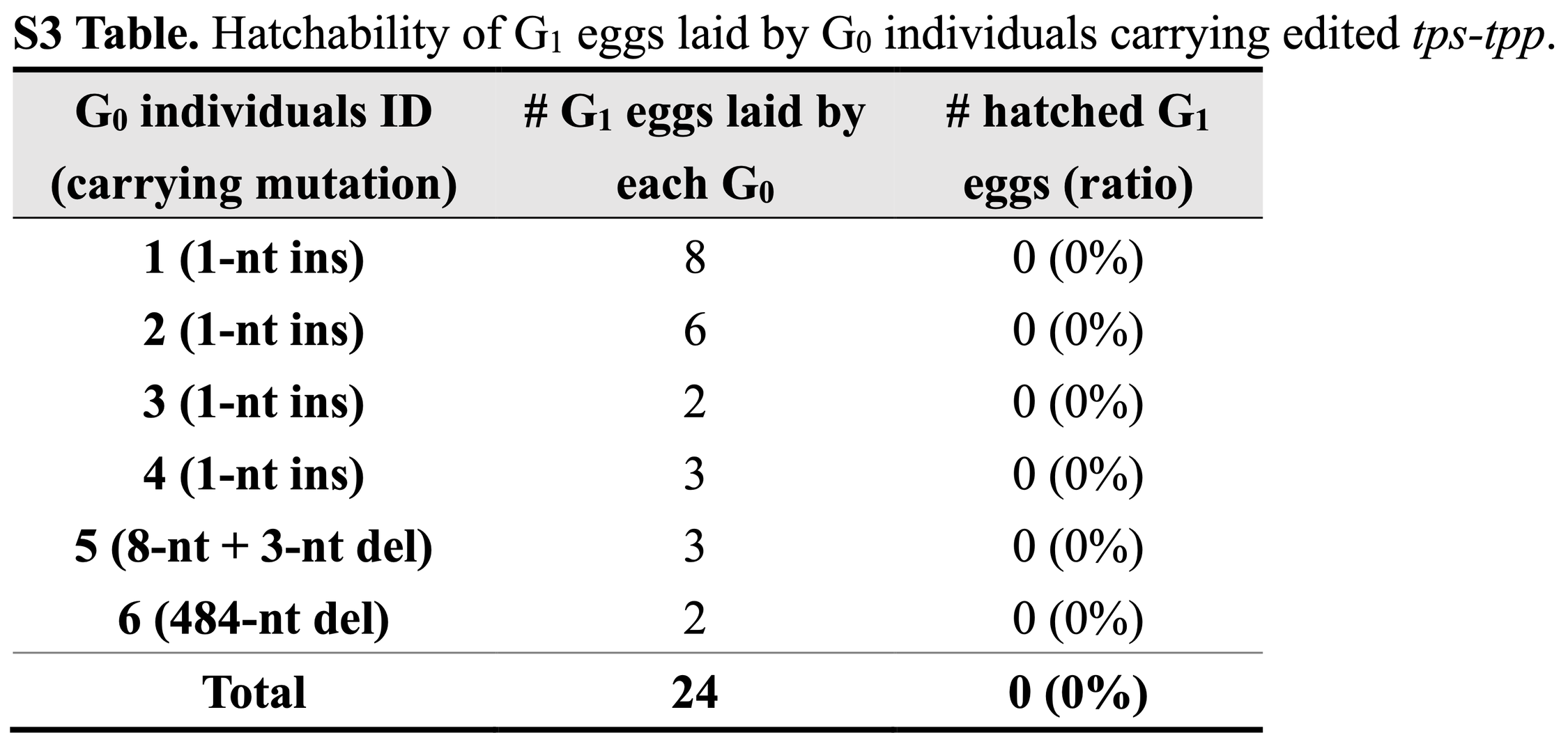

Supplement: S3 Table — (TIF) [file pgen.1011298.s011.tif]

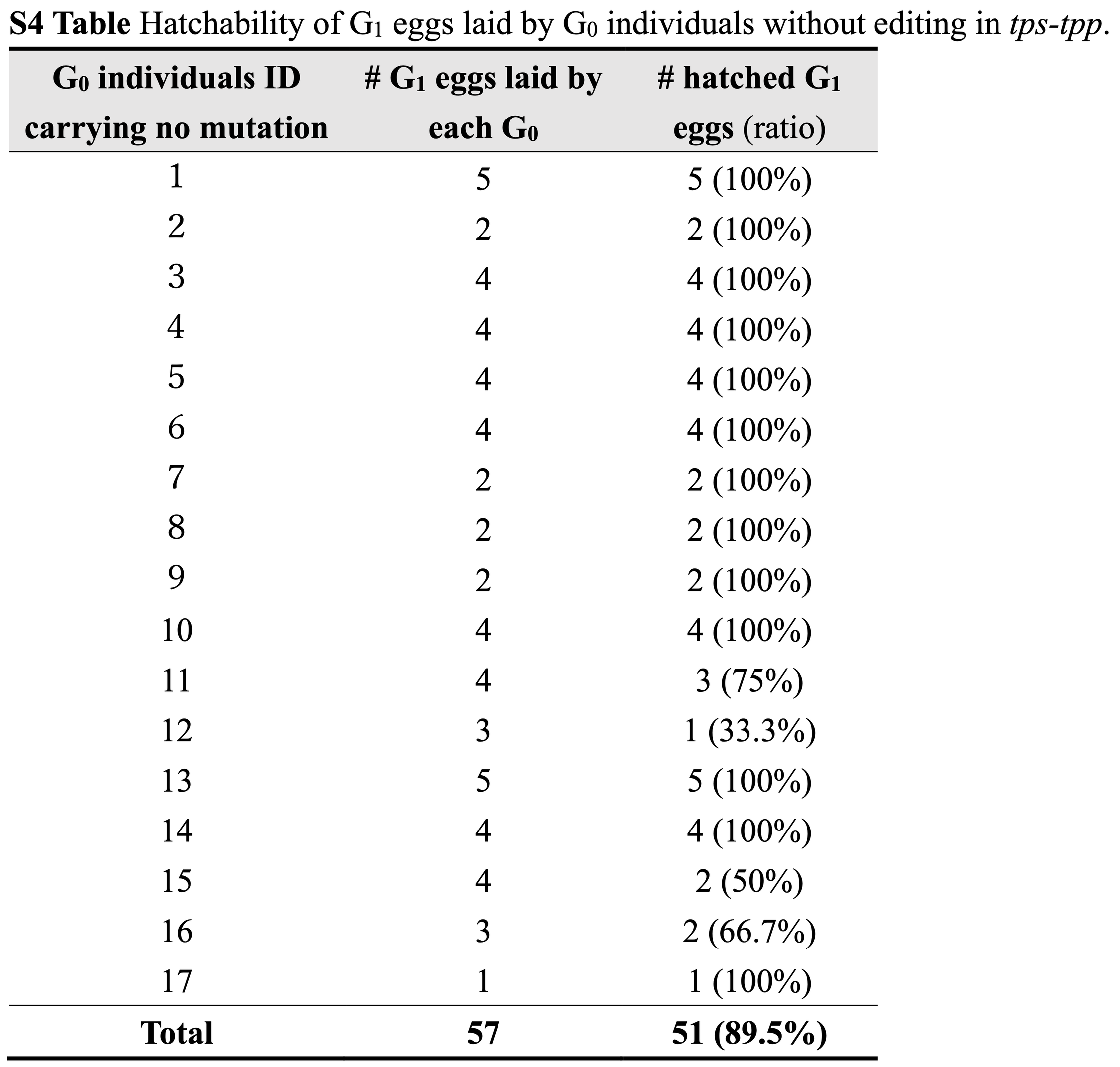

Supplement: S4 Table — (TIF) [file pgen.1011298.s012.tif]

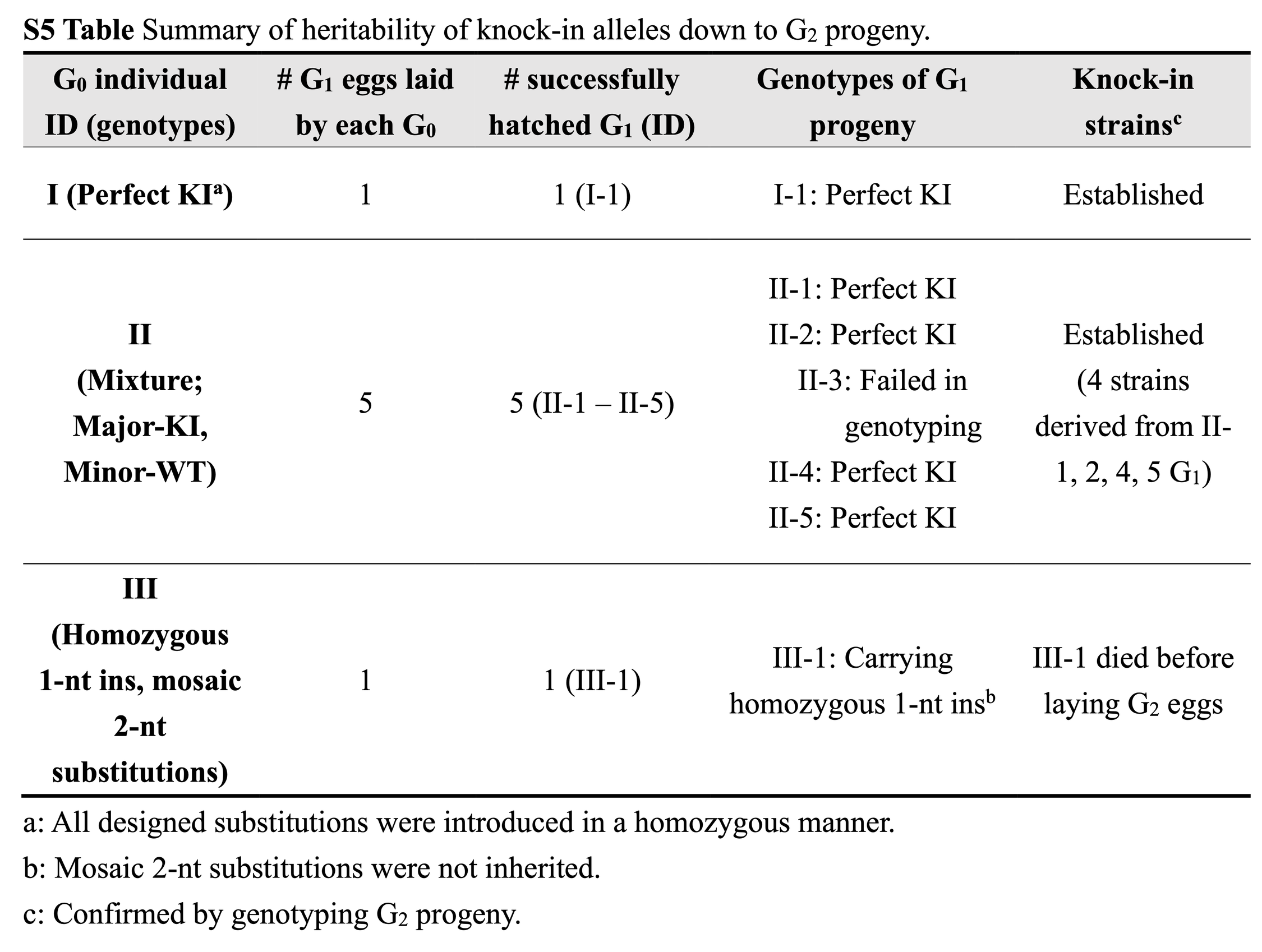

Supplement: S5 Table — (TIF) [file pgen.1011298.s013.tif]

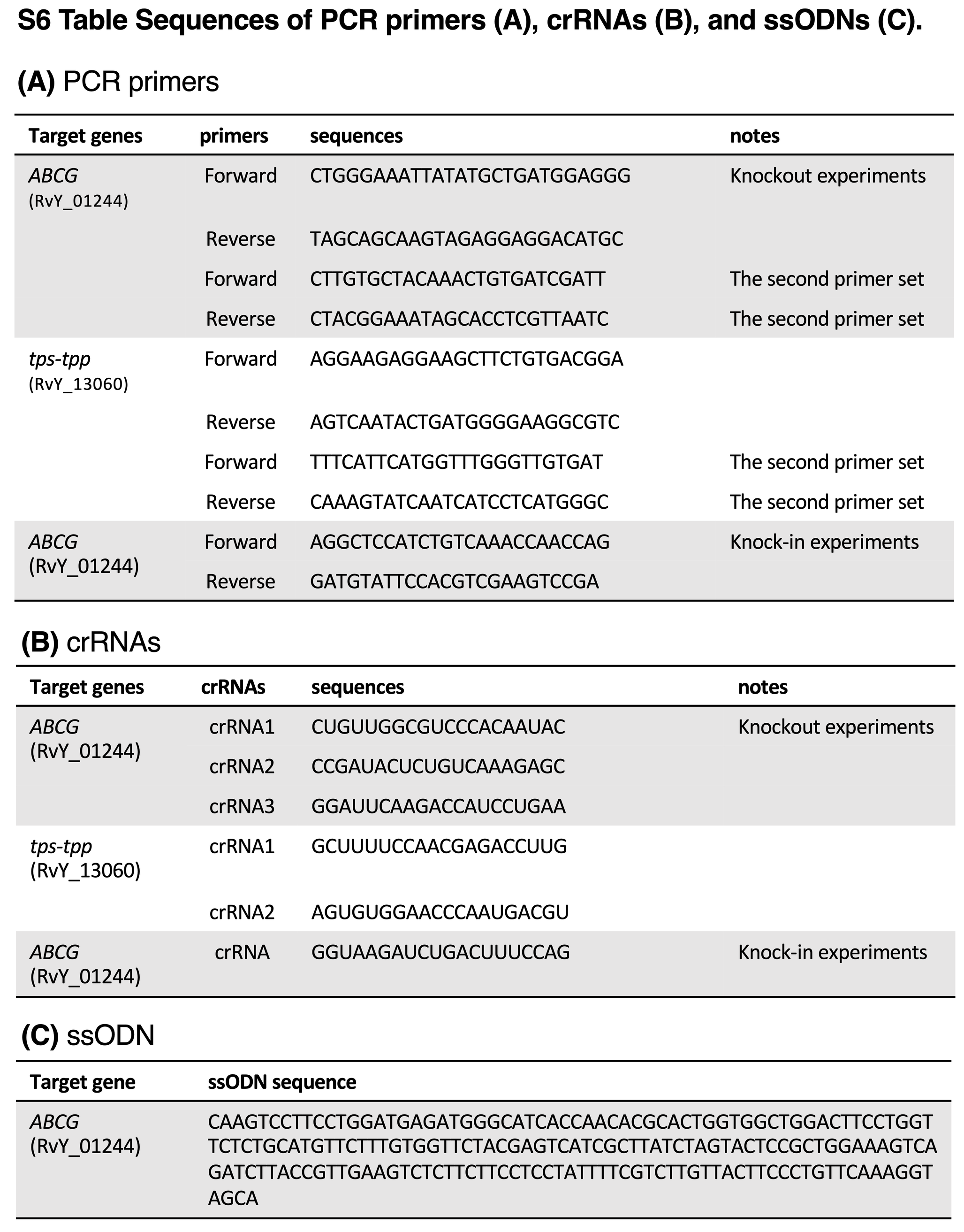

Supplement: S6 Table — (TIF) [file pgen.1011298.s014.tif]
