# Supplementary material for: Single-step generation of homozygous knockout/knock-in individuals in an extremotolerant parthenogenetic tardigrade using DIPA-CRISPR
Source: PLoS Genet. 2024 Jun 13;20(6):e1011298. doi: 10.1371/journal.pgen.1011298 (PMC11175437; doi:10.1371/journal.pgen.1011298)
Supplement: Attachment — Submitted filename: Response_to_comments20240418clean.docx [file pgen.1011298.s016.docx]

**Responses to the comments:**

**Comments from the Associate Editor:**

As you will see below, the comments of the three reviewers are split: #1 and #3 are basically satisfied with the novelty and significance of this study, but #2 is not. I would like to suggest that the authors revise the manuscript according to the reviewers’ comments, with a stronger emphasis on the importance of tardigrade biology and the significance of this work within it.

Thank you for the helpful and thoughtful comment. We have added sentences emphasizing the importance of tardigrade biology (L63-L72, L112-L117) and the significance of this work (L109-L111) as follows.

(L63-L72)

“tardigrades can be stored in a desiccated state at room temperature, sometimes for over a decade [3]. Dehydrated tardigrades exhibit extraordinary resilience against various extreme stresses that would kill most other animals, such as low temperature (−273 °C), intense irradiation, the vacuum of space, and high hydrostatic pressure (7.5 GPa) [4-8]. This resilience is believed to arise from their remarkable cellular protection and repair mechanisms, which safeguard essential biomolecules and structures such as DNA, RNA, proteins, and membranes, supporting cellular functions. Understanding the molecular players and mechanisms involved in these processes not only satisfies scientific curiosity but also holds promise for the development of innovative technologies with significant implications for the storage and distribution of valuable but fragile biomaterials, like biomedicines and vaccines. Despite the growing interest in tardigrade resilience, the molecular mechanisms underlying this resilience have remained largely elusive.”

(L112-L117)

“In addition to their renowned resilience, tardigrades are becoming increasingly recognized as an emerging model for evolutionary and developmental biological study [28]. This is because the phylum Tardigrada has a close taxonomic relationship to two phyla containing super model invertebrates, *Drosophila melanogaster* (phylum Arthropoda) and *Caenorhabditis elegans* (phylum Nematoda), and would be expected to comprise suitable organisms for comparative study among them. Our method will also open up avenues for studying various Evo-Devo topics using tardigrades.”

(L109-L111)

“This is the first example of heritable gene editing in the entire phylum of Tardigrada, and this gene-editing method should substantially promote *in vivo* analysis of the molecular mechanisms underpinning the extreme tolerance of tardigrades.”

These emphases have been repeated in the Abstract, Author summary, and Discussion as well.

1. As pointed out by the reviewers, the language of this manuscript should be extensively improved.

We apologize for the inadequate English in the previous version of the manuscript. To improve the writing, our revised manuscript has been copyedited by a professional service. We hope that the language of the revised manuscript is now satisfactory.

2. As pointed out by reviewer #3, a weakness of this paper would be the lack of in-depth functional studies of the target genes. However, since this paper is submitted to the Methods section/category, additional functional studies on a different/new target gene would not be necessary. Instead, I would suggest that the authors discuss more about the results of the gene editing experiments. For example, it is not clear whether the authors found some external phenotypes in the RvY_01244 KO animals. It is also unclear whether this gene is an ortholog of insect white genes. As insects have multiple white homologs (e.g., scarlet and brown in Drosophila, which form a heterodimer with white), and some species have multiple copies of white paralogs, the authors should carefully validate the characteristics of RvY_01244 and whether it is a tardigrade white gene.

Thank you for the thoughtful comments. Accordingly, we have performed phylogenetic analysis of ABCG family genes, such as seven tardigrade proteins including RvY_01244 and 59 ABCG proteins from four model insects. The results suggested that RvY_01244 is not orthologous to white and thus we replaced all expressions of “*white*” with “ABCG” while presenting the phylogenetic analysis as the new S1 Fig. We have also described the phenotype of the m1–m3 mutants of RvY_01244 (formerly *w*-m1 to *w*-m3), some of which exhibited significant growth retardation including growth arrest before the initiation of pigmentation (L195-L205). However, similar phenotypes were observed in G_0_ siblings without edits, and we failed to notice the m1–m3 mutants before genotyping and did not have photographs of them. Therefore, we carried out additional KO experiments targeting RvY_01244 and obtained a new KO mutant termed m4 with photographs. The m4 mutant and most siblings with no edits exhibited significant growth retardation, but both exhibited brown coloration. We have presented the associated data in Fig. 2F and S3 Fig. We have also described the m4 mutant in L195-L205. Moreover, we have added photographs and a description of *RvY_01244* knock-in individuals in S7 Fig. and L331-L332.

3. For readers not familiar with tardigrades, it would be nice if the authors could show the pictures of tardigrades being injected or oocytes/eggs being developed or laid.

Thank you for this helpful comment. We have added pictures of eggs and adults of *R. varieornatus* in Fig. 1A–C.

**Responses to Reviewers’ Questions**

Comments to the Authors:

Please note here if the review is uploaded as an attachment.

**Reviewer #1:** In this manuscript, Kondo et al demonstrate germline CRISPR (i.e., heritable edits) for the first time in the tardigrade phylum. The Kunieda lab had previously developed CRISPR in somatic cells, and here they have tried to extend this to heritable, germline edits by injecting Cas9 RNPs into the body cavity of tardigrades during late oogenesis, as has worked in insects. They first injected glycerol at various concentrations as a precursor to injecting a commercial Cas9 in glycerol, and they showed that about half of injected animals survive a concentration of glycerol that enables a similar concentration of Cas9 to be injected as was used in insects. Then they injected Cas9, tracRNA and crRNA as an RNP complex, and showed that injecting 8-10 days after hatching resulted in gene edits. This worked targeting a second gene as well, and they showed multiple kinds of edits, some homozygous edits, and inheritance of edits 1-2 generations later. Lastly, they showed that co-injecting single-stranded oligo DNA could be used to knock in at least short sequences.

The development of germline CRISPR (i.e., heritable edits) is a big step forward for an emerging model organism, and this had never been done before for any tardigrade (an entire phylum of animals). And there's broad interest in the use of tardigrades – arguably the animal kingdom's most extreme survivor – for understanding how biological materials can survive extremes. The experiments appear well thought out and reported clearly. I'm thrilled to see the development reported here, and I have only minor concerns.

Minor concerns:

1. The number of animals genotyped suggests that PCR often failed, which gives me just a little concern about whether PCR failures could have resulted in apparent homozygosity in what were actually heterozygotes. This concern could be taken care of by re-PCRing from some of the samples using a few pairs of primers (instead of just one primer pair) and seeing if the results are the same (and moving primers further out, to amplify longer sequences, would reveal whether edits can be found a little further out from the targeted sites).

Thank you for the careful comments. We agree that it would be better to check the homozygosity of the edits by repeating PCR with a different primer pair. We have carried out those experiments for four edited samples (of ABCG, formerly white, and tps-tpp editing) and confirmed the homozygosity of those edits. We have presented these data in S2 and S5 Figs and have described the results in L172-L173 for *ABCG* KO trials and in L278-L280 for *tps-tpp* KO trials.

2. The manuscript needs English editing, and I would argue would benefit tremendously from a good scientific copy editor. The ideas expressed are interesting, and I found the Discussion especially thoughtful and articulate, but the mechanical issues in the writing will be an obstacle for many readers.

Thank you for the positive and helpful comments. We apologize for the substandard English in the previous manuscript. To improve the writing, our revised manuscript has been copyedited by a professional service.

3. Hypsibius exemplaris is described as having relatively weak tolerance ability. Hypsibius exemplaris requires slow drying to survive desiccation, and this often fails, but once in tun state, I'm not aware of any studies showing that they are significantly less resistant than other tardigrades to extremes. And Hypsibius exemplaris survives irradiation as well as other resistant tardigrades do. I think the authors mean to say that Hypsibius does not survive rapid desiccation, and not that their tolerance abilities are weak.

In response to your comments, we have deleted the description “whose tolerant ability is relatively weak among anhydrobiotic tardigrades” (L81-L82 in the revised version of the manuscript). We think the detailed description of a characteristic of *H. exemplaris* tolerance is unnecessary here.

4. p. 13, line 275: The supplementary figure cited does not show the relevant data. Also, statements are made at the end of this paragraph without data shown.

The text had claimed that G_2_ progeny inherited the perfect KI allele from the G_0_ individual in a homozygous manner, and the corresponding supplementary figure (S6 Fig. in the revised manuscript) showed the Sanger sequencing data of one G_1_ and three G_2_ progeny derived from the homozygous knock-in G_0_ individual, all of which carried the perfect knock-in allele. We think the data provided evidence for the heritability of the knocked-in allele to G_2_ progeny. To clarify this, we have rephrased the sentence as “From a perfectly knocked-in G_0_ individual, one G_1_ progeny was successfully reared and propagated, in which all of the examined G_1_ and G_2_ progeny were confirmed to carry the perfectly knocked-in allele in the homozygous form (S6 Fig. and S5 Table).” in L329-L331. We have also added a new S5 Table providing a summary of genotyping down to the G_2_ generation.

5. p. 14, line 297: This paper speculated that storage cells synthesize yolk, but to my knowledge this has never been demonstrated (and there is not yet a marker for yolk protein, to my knowledge).

In a previous TEM study (*), significant accumulation of reserve materials was observed in storage cells during the early/middle stage of vitellogenesis; this accumulation rapidly decreased in the subsequent phase. Accordingly, storage cells were proposed as parts of tissue synthesizing yolk(-precursor) materials (*). However, as commented by the reviewer, we agree that there is no solid evidence for this assumption. Therefore, we removed “(in storage cells)” in L353. Genes encoding vitellogenin homologs are found in the genome in both *R. varieornatus* and *H. exemplaris*, and their expression generally increases in the adult stage, although no data about their function and localization have been reported.

(*)

Hyra M *et al*., Body cavity cells of Parachela during their active life. *Zool J Linn Soc* 2016; **178**: 878-887.

<https://academic.oup.com/zoolinnean/article/178/4/878/2691472>

6. p. 15, line 328: Comparing knock-in and knock-out efficiency: Has a statistical test been done to see if this is a real difference? I suggest doing an appropriate test and then describing the two efficiencies as either different or indistinguishable.

In accordance with this comment, we performed Fisher’s exact test, by which no significant differences were detected (p=0.77, all KO vs. ABCG KI; p=1.0, ABCG KO vs. ABCG KI). Therefore, we have removed “even slightly higher than” (L327-L328 in the previous version of the manuscript) and have modified the statement as follows: “knock-in efficiency was comparable to that of knockout” (L389).

**Reviewer #2:** Kondo et al. provide a first report on gene-edited GO progeny of tardigrades using the DIPA-CRISPR technique. They created knockouts of the white and tps-tpp gene, and also created a knock-in for the white-gene using ssODNs. Materials and methods are clear, and Discussion is to the point. I did not find any major flaws in the manuscript (see some comments below). However, as the CRISPR technique that was used to genetically transform tarfigrades is largely based on a previously published method (Shirai et al. 2022, Cell Reports), the research in the manuscript can, in my opinion, not be considered as a major advancement in the field and, hence, might better fit in another journal than PLoS Genetics.

Thank you for the insightful comments. We believe that our manuscript presents three significant advancements. (1) First, as highlighted by reviewer #1, our work marks the pioneering development of heritable genome editing for the first time within the entire Tardigrada phylum. The considerable increase in publications on molecular analysis in tardigrades, following the identification of several tardigrade-unique protective proteins, underscores the growing interest in tardigrade resilience and potential applications. The most important component missing in tardigrade research was a technology for heritable gene editing. Our study has effectively addressed this longstanding challenge in the field. (2) Second, we provided evidence demonstrating the broad applicability of DIPA-CRISPR beyond insects. Prior to our study, DIPA-CRISPR had only been tested in insects, with no reported data even on non-insect arthropods. Our findings established the effectiveness of DIPA-CRISPR in a phylum outside of insects, expanding its utility. (3) Third, we presented evidence that a simple injection predominantly yields homozygous diploid mutants, rather than hemizygous haploid mutants (please see the response to the comment below for details), as G_0_ progeny, particularly in the parthenogenetic tardigrade *R. varieornatus*. This discovery sheds light on the generation of homozygous mutants through similar methods in parthenogenetic species.

Considering these contributions, we believe that our manuscript would be suitable for publication in this journal.

Comments:

line 59: In a previous study,...

Thank you for pointing this out. After copyediting by a professional service, we have omitted this part that was in the previous version of the manuscript (L81).

line 124: it is not clear to me whether RvY_01244 is an 1:1 ortholog of Drosophila white, or does the tardigrade has multiple white paralogs? Authors should include a ABCG phylogeny to demonstrate orthology

Thank you for this helpful comment. We apologize for our previous ambiguous description of RvY_01244, which may have been misleading. We meant that RvY_01244 belongs to the ABCG family, that is, one of the ABCG transporters, not that it is a 1:1 ortholog of *Drosophila white*. In accordance with the reviewer’s suggestion, we performed a phylogenetic analysis of 66 ABCG proteins from the tardigrade (*Ramazzottius varieornatus*) and four model insect species. The tardigrade has seven ABCG transporters. The obtained tree topology is generally consistent with the previous literature on insect ABCGs (*). While two tardigrade ABCG proteins (not RvY_01244) exhibited a close relationship with the *Drosophila* White protein, no clear orthologs were found for Scarlet and Brown in the tardigrade. RvY_01244 was shown to be closely related to Atet (ABC transporter expressed in trachea; another ABCG protein) of *Drosophila*, suggesting that RvY_01244 is one of the ABCG transporters (possibly Atet), but not an ortholog of *Drosophila white*. Accordingly, we have replaced all descriptions of “white” with “ABCG” throughout the manuscript and described the result of the phylogenetic analysis in L152-L155.

Our aim in the study reported here is to develop a germline CRISPR method and any gene can be a target as far as mutations (genome editing) can be detected. Therefore, we believe that this change of description does not affect the meaning and value of our manuscript. Thank you again for providing valuable comments to remove ambiguous descriptions and clearly improve our manuscript.

(*)

Dermauw W and Van Leeuwen T. The ABC gene family in arthropods: comparative genomics and role in insecticide transport and resistance. *Insect Biochem Mol Biol*. 2014; **45**: 89-110. doi: 10.1016/j.ibmb.2013.11.001.

<https://www.sciencedirect.com/science/article/pii/S0965174813001951>

line 129-135: it is not clear to me what the phenotype is of w-m1, w-m2 and w-m3, this should be mentioned in the results section (including photographs of the w mutants)

Thank you for this comment. We have renamed the mutants m1–m3 because RvY_01244 is not orthologous to white. The mutants m1 and m2 (both of which likely suffered disruption of protein function due to a frameshift near the N-terminus) exhibited growth arrest before coloration started, whereas the m3 mutant (carrying a 1-amino-acid deletion without a frameshift) appeared normal. Unexpectedly, many G_0_ siblings carrying no edits exhibited significant growth retardation, so it was difficult to specify a mutation-specific phenotype. This is part of the reason why we failed to notice these mutants before genotyping and did not take photographs of m1–m3. Therefore, we carried out additional RvY_01244 gene-KO experiments while photographing all G_0_ individuals before genotyping and obtained another RvY_01244 KO mutant termed m4, which carried frameshift mutations near the N-terminus. This mutant m4 again exhibited significant growth retardation, but its body color became pale brown, which was indistinguishable from the siblings carrying no edits, suggesting that RvY_01244 is not responsible for brown coloration. We have added the photographs of the m4 mutant and its sibling with no edits (S3 Fig.) along with a description of the phenotype of all mutants (m1–m4 and knock-in) in the Results section (L195-L205; KI, L331-L332, S7 Fig.) and Discussion section (L395-L405).

line 176: as with the white gene; authors should construct a phylogenetic tree, displaying orthology between tardigrade tps-tpp and tps-tpp of other species; In addition, is the tps-tpp gene of R. varieorantus horizontally transferred? If so, make this more clear in the results section.

The *tps-tpp* (trehalose synthesis) gene exhibits a scattered distribution in the animal kingdom. Our previous study thoroughly analyzed its phylogenetic relationships and revealed a complicated evolutionary history of this gene (*). Specifically, a common ancestor of animals had acquired the *tps-tpp* gene (designated as pan-metazoan *tps-tpp*), but this gene is currently retained in only a few taxa/species (i.e., arthropods and a limited number of species in cnidarians, echinoderms, amphioxus, tunicates, and monogonont rotifers). Most other species (other than arthropods) have lost this gene, but two lineages of tardigrades including *Ramazzottius varieornatus*, nematodes, and bdelloid rotifers have independently acquired the distinct bacterial *tps-tpp* gene via horizontal transfer. Through extensive validations, our previous study also confirmed that these horizontally transferred genes are not derived from contaminants. We think that it is inadequate to repeat the same analyses of our previous study in this manuscript. Instead, we more clearly described the results of phylogenetic analysis in L222-L225 while retaining the citation of our previous publication.

(*)

Hara *et al*., Parallel evolution of trehalose production machinery in anhydrobiotic animals via recurrent gene loss and horizontal transfer. *Open Biol*. 2021, **11**(7): 200413.

<https://royalsocietypublishing.org/doi/10.1098/rsob.200413>

line 316: ... undergoes a..

Thank you for pointing this out. Upon copyediting by a professional service, we have omitted a/the in the previous version of the manuscript (L372).

line 319/320: mutants of haplodiploid insects/arthropods can also be obtained in a single step, see e.g. 10.1016/j.ibmb.2023.104068 (spider mites and thrips) or 10.1089/crispr.2019.0067 (whiteflies); authors should integrate these studies in the Discussion section

Thank you for letting us know about these important and fascinating publications. We agree that, in haplodiploid insects/arthropods, a haploid individual (usually male) has a single allele, but we suppose that this situation is usually described as “hemizygous” rather than “homozygous”. In addition, to establish a homozygous strain from the obtained hemizygous male, multi-step (sometimes complicated) crossings and selections are required. To clarify the difference between this study (homozygous parthenogenetic mutant) and a hemizygous haploid mutant in haplodiploid insects/arthropods, we have described the comparison with the case of haplodiploid species in the Discussion section (L377-L382).

**Reviewer #3:** The current study by Kondo et al explores the use of gene editing techniques, specifically CRISPR-Cas9 and DIPA-CRISPR, in tardigrades, microscopic organisms known for their extreme resilience. The authors report the successful application of gene editing methods to modify genes in tardigrades, in particular with focus on the anhydrobiotic and extremotolerant species Ramazzottius varieornatus. They adapted the Direct Parental CRISPR (DIPA-CRISPR) method, initially developed for insects, to inject Cas9 ribonucleoproteins (RNPs) into female tardigrades. This resulted in gene-edited progeny, demonstrating the method's effectiveness. The study also explored gene knock-in trials with single-stranded oligodeoxynucleotides (ssODNs), achieving notable efficiency. This research provides significant insights into the molecular mechanisms of tardigrades' resilience and opens new avenues for functional genomics research in these organisms.

Overall, I find the subject of the manuscript to be interesting and relevant. The paper is for the most part well written; however, the manuscript would benefit from more careful copy editing, as there are many instances of wrong word-/verb-use making it difficult to understand what the authors mean. My perhaps biggest critique is that this is largely a descriptive methods paper, rather than a detailed functional study, which is otherwise expected by a PLoSGen paper. However, given the novelty and difficulty in performing genome editing in these animals, this is perhaps to be expected. Nevertheless, I would encourage the authors to consider adding more functional data illustrating the utility of this approach (see below), as the whole methodology argues that this would be entirely feasible.

Thank you for the comments. We apologize for the many instances of incorrect word/verb use in the previous version of the manuscript. To improve the writing, our revised manuscript has been copyedited by a professional service. As for functional study, we describe our responses below (in response to the major comments).

Major comments:

1. Given that the authors report that this tardigrade species reproduce parthenogenetically, I was wondering if they allowed the w-m1-3 mutants to lay eggs prior to harvesting their DNA for sequencing (if there are no WT alleles present I assume the mutation was introduced in the germline, as one would otherwise observe a mosaic pattern)? Are these mutant lines still available, and if so, did the authors report any phenotypes associated with gene mutation, i.e. pigment defects or similar? I see later on that the authors did in fact attempt to propagate the F1 generation following their knock-in protocol, suggesting that this is a viable approach. However, again, there are no attempts made of performing phenotypic characterization.

Thank you for your thoughtful comments. The mutants m1–m3 (formerly *w*-m1 to *w*-m3) had been subjected to genotyping before laying G_1_ eggs, so we do not currently have those mutant lines. In the revised version of the manuscript, we present the phenotype of these mutants; specifically, m1 and m2 (both of which likely suffered the disruption of protein function via a frameshift near the N-terminus) exhibited growth arrest before coloration started, whereas the m3 mutant (carrying a 1-amino-acid deletion without a frameshift) appeared normal. Unexpectedly, many G_0_ siblings carrying no edits exhibited significant growth retardation, so it was difficult to specify the mutation-caused phenotype. This explains why we failed to notice these mutants before genotyping and did not take photographs of m1–m3. Therefore, we carried out additional RvY_01244 gene-KO experiments with photographing of all G_0_ individuals before genotyping and obtained another RvY_01244 KO mutant termed m4, which carried frameshift mutations near the N-terminus. This m4 mutant again exhibited significant growth retardation, but its body color became pale brown, which is indistinguishable from the siblings carrying no edits. This suggests that RvY_01244 is not responsible for the brown coloration. We have added photographs of the m4 mutant and its sibling with no edits (S3 Fig.) and a description of the phenotypes of all mutants (m1–m4 and knock-in) in the Results section (L195-L205; KI, L331-L332, S7 Fig.) and Discussion section (L395-L405).

If the authors could show, either 1) by knock-out of a non-essential gene followed by in-depth phenotypic characterization, or alternatively 2) by knock-in of e.g. GFP under control by a promotor of choice, it would significantly elevate the utility and impact of this paper.

In the phylum Tardigrada, most species do not have the trehalose synthesis gene (*tps-tpp*) and only two lineages including *R. varieornatus* have acquired the bacterial *tps-tpp* gene, indicating that the *tps-tpp* gene is not generally essential for tardigrade physiology. However, as presented in the manuscript, we showed that mutation in the *tps-tpp* gene had a maternal effect on the hatchability of eggs, although this is the first example of clear phenotypic characterization of a gene mutation in the entire phylum of Tardigrada. At this point, knowledge on the molecules/genes in the phylum Tardigrada is quite limited and it is not a simple task to identify non-essential genes in this phylum, as suggested in the reviewer’s comment. Our established method should facilitate elucidation of the roles of various molecules/genes in this phylum and provide a powerful tool to enable the analysis suggested by the reviewer in future work. This manuscript has been submitted to the Methods category of the journal, and we believe that the method we have described provides a sufficiently significant step forward, enabling various analyses including those suggested by the reviewer.

Minor comments:

L32 Do the authors mean “conducive” instead of “conductive”?

Thank you for the correction. You are right. We have replaced “conductive” with “that led to” (L26)

L59-63 Consider revising this sentence as it is long and difficult to follow.

We have divided the sentence into two sentences to improve readability (L81-L84).

L68 “flour” instead of “floor”

Thank you for pointing this out. We have corrected it. (L89)

L124 I assume the authors mean that RvY_01244 is predicted to encode a homologue of Drome-white? What is the degree of sequence homology between these two genes? To what extent are the core functional motifs of this ABCG2 transporter conserved, i.e. what is the data supporting that these genes are homologous? It could be interesting to show how this famous gene has changed over the course of evolution.

Thank you for providing this important comment. We apologize for our previous ambiguous and misleading description of RvY_01244. We meant that RvY_01244 belongs to the ABCG family (also known as the White subfamily of ABC transporters); that is, RvY_01244 is one of the ABCG transporters, but not necessarily a 1:1 ortholog of *Drosophila white*. In the revised manuscript, to clarify the relationship between RvY_01244 and *Drosophila white*, we have presented a phylogenetic analysis among seven ABCG transporters of the tardigrade (*Ramazzottius varieornatus*) including RvY_01244, and 59 ABCG transporters from four model insect species including *Drosophila* White. The obtained tree topology is generally consistent with the previous literature on insect ABCGs (*). While two tardigrade ABCG proteins (not RvY_01244) exhibited a close relationship with *Drosophila* White protein, no clear orthologs were found for Scarlet and Brown in the tardigrade. RvY_01244 was closely related to Atet (ABC transporter expressed in trachea; another ABCG protein) of *Drosophila*, suggesting that RvY_01244 is one of the ABCG transporters (possibly Atet), but not an ortholog of *Drosophila white*. Accordingly, we have replaced all descriptions of “white” with “ABCG” throughout the manuscript and described the result of the phylogenetic analysis in L152-L155.

Our aim in the work described in this manuscript is to develop a germline CRISPR method and any gene can be a target as far as mutations (genome editing) can be detected. Therefore, we believe that this change of description does not affect the meaning and value of our manuscript. Thank you again for providing valuable comments to remove ambiguous descriptions and clearly improve our manuscript.

(*)

Dermauw W and Van Leeuwen T. The ABC gene family in arthropods: comparative genomics and role in insecticide transport and resistance. *Insect Biochem Mol Biol*. 2014; **45**: 89-110. doi: 10.1016/j.ibmb.2013.11.001.

<https://www.sciencedirect.com/science/article/pii/S0965174813001951>

L130 Add “for” after “amplicons”.

Thanks for this advice. We have inserted “for” after “amplicons.” (L161)

Figures.

Figure 1 You mention in the result section that you examine injection into animals that are 5-10 days old, yet the figure shows 7-10 days. Which one is it? Please correct text and figure accordingly.

We have corrected this from “7 – 10 d” to “5 – 10 d” in Figure 1. We also added a note to the figure legend that gene-edited G_0_ progeny were obtained from injected 7- to 10-day-old parents.
