# Supplementary material for: Single-step generation of homozygous knockout/knock-in individuals in an extremotolerant parthenogenetic tardigrade using DIPA-CRISPR
Source: PLoS Genet. 2024 Jun 13;20(6):e1011298. doi: 10.1371/journal.pgen.1011298 (PMC11175437; doi:10.1371/journal.pgen.1011298)
Supplement: Attachment — Submitted filename: response-rev2-20240509-KK.docx [file pgen.1011298.s017.docx]

**Responses to the comments:**

**Reviewer #1**: I am content with all of the edits that the authors made in response to my concerns. The development of heritable CRISPR-based genome editing is a big step forward for a phylum where it had never been done before and will open up studies that are not possible in other organisms, given tardigrades' unique ability to survive some remarkable extremes.

Thank you for encouraging comments.

**Reviewer #2**: The authors addressed most of my comments (e.g. ABCG orthology) in an adequate way, and made more clear that their study addressed a longstanding challenge in the field (i.e. heritable gene editing of tardigrades). However, I am not completely satisfied with all replies. Please find a below some remarks to their responses:

1. In their reply to my first comment the authors state that DIPA-CRISPR has only been tested in insects. This seems not correct as in Dermauw et al. 2020 adult females of the spider mite T. urticae (Arthropoda:Chelicerata) were injected with Cas9/sgRNAs and KO mutants were succesfully generated. It was only in 2022, that this technique was termed DIPA-CRISPR by Shirai et al. 2022. Authors should correct their manuscript accordingly.

Thank you for letting us know the important information. In accordance with the reviewer’s comment, we modified the sentence as below.

L431-433 (Blue indicates modifications)

While DIPA-CRISPR and similar methods were initially developed in arthropods [27, 43], our study shows its effectiveness in a non-arthropod organism, underscoring the broad applicability of this method to various invertebrate species,

43. Dermauw W, Jonckheere W, Riga M, Livadaras I, Vontas J, Van Leeuwen T. Targeted mutagenesis using CRISPR-Cas9 in the chelicerate herbivore *Tetranychus urticae*. Insect Biochem Mol Biol. 2020; 120: 103347.

2. description of "mutant phenotypes": what is the difference between growth arrest (m1, m2) and growth retardation (m4)? Authors should be more clear.

Thank you for pointing out the ambiguity. We did not observe any distinct brown coloration in m1 and m2 and thus we had used the word “growth arrest”. However, we sacrificed m1 and m2 for genotyping slightly earlier than m4 mutant and thus the discrimination between two terms, “growth arrest” and “growth retardation” was ambiguous. Therefore, we combined these into the term “growth retardation” and have added a more detailed explanation as below (L196-199, L205, L399).

L196-199 (Blue indicates modifications)

Among four obtained *RvY_01244* mutants, three of them, m1, m2, and m4, exhibited significant growth retardation. Two of them, m1 and m2 were sacrificed for genotyping at 10 and 12 days old, respectively, when they were very small and their body color was barely visible, while m4 was cultured for an extended period until 16 days old, at which point its body color became brown though its body was still much shorter than usual (S3 Fig. A).

L205 and L399

We have also replaced the word “arrest/retardation” with “retardation”.

3.L379/L380: "In these cases, multiple crossings and selections are needed to establish homozygous mutant strains". This statement is not correct as in a recent study (10.1016/j.ibmb.2023.104068) it was shown that homzygous mutants could be generated via Cas9/sgRNA injection of fertilized females. Authors should rephrase.

Thank you for letting us know the possible homozygous diploid mutation via SYNCAS which is a new method utilizing BAPC and saponin to improve the efficiency of DIPA-CRISPR. The indicated publication has already been cited in our previous manuscript and thus we carefully rechecked the publication. The publication demonstrated that the application of SYNCAS to fertilized female mites produced some diploid progenies (F1 females) exhibiting a recessive KO phenotype suggesting biallelic functional mutations (Fig. 3B in the publication). However, sequencing analyses of the following F2 male (haploid) progenies revealed that every F1 female with recessive KO phenotype carried 2 or 3 distinct mutant alleles without WT allele (Fig. S2). These data indicated that all of the presented F1 females were biallelically mutated but not homozygous. This is consistent with the decreased proportion of KO phenotype in diploid female than those in haploid male, suggesting that multiple mutations in diploid genome occurred independently (Fig. 3B). These circumstances are much different from those in our publication where most of mutated females (parthenogenetic diploid) carried single mutant allele, i.e., homozygous. Therefore, we do not think it is needed to rephrase here.

**Reviewer #3**: The authors have addressed all concerns raised successfully and I have no further comments.

Thank you for having provided constructive comments.
